# The complete chloroplast genome sequence of *Polypodiodes amoena* (Polypodiaceae), an important medical fern

**DOI:** 10.1080/23802359.2019.1642154

**Published:** 2019-07-18

**Authors:** Ziqi Kang, Xinyao Sun, Yanling He, Ziqing He, Yongfeng Hong, Zhen Wang, Ting Wang, Yingjuan Su

**Affiliations:** a School of Life Sciences, Sun Yat-sen University, Guangzhou, China;; b College of Life Sciences, South China Agricultural University, Guangzhou, China;; c Research Institute of Sun Yat-sen, University in Shenzhen, Shenzhen, China

**Keywords:** *Polypodiodes amoena*, chloroplast genome, phylogenetic analysis

## Abstract

*Polypodiodes amoena* is an important medical fern of Polypodiaceae. Its complete chloroplast genome is obtained through Illumina sequencing, which is 152,067 bp in length with a large single copy (LSC) region (81,187 bp), a small single copy (SSC) region (21,590 bp), and two inverted repeats (IRa and IRb) regions (24,645 bp). The genome encodes 130 genes, including 88 protein-coding genes, 33 tRNA genes, eight rRNA genes, and one pseudogene. ML phylogenetic analysis reveals that *P. amoena* is clustered with polypodiaceous ferns.

*Polypodiodes amoena* (Wallich ex Mettenius) Ching is an important medical fern of Polypodiaceae known as ‘Gusuibu’ for relieving rigidity of muscles and activating collaterals, and clearing heat and detoxication (National Pharmacopoeia Committee 2010). As a medicinal part, its rhizome is 5–7 mm in diameter, densely covered with dark-brown scales. The plant is epiphytic growing on rocks or on tree trunks with a wide distribution in south China such as Guangdong, Guangxi, and Guizhou, etc. (Zhang et al. 2013). In addition, *Polypodiodes* is a core group for investigating the controversial phylogenetic relationship among *Polypodiastrum*, *Schellolepis*, and *Goniophlebium* (Schneider et al. 2004; Lu and Li 2006). Therefore, the sequencing of the whole chloroplast genome of *P*. *amoena* lays a solid foundation for further exploring phylogeny of the family Polypodiaceae.

The fern was provided by South China Botanical Garden, Chinese Academy of Sciences (23°11′3.56″N, 113°21′43.28″E). The specimen is held by Herbarium of Sun Yat-sen University (SYS; voucher: SS Liu 20161021). The total genomic DNA was isolated from fresh leaves using Tiangen Plant Genomic DNA kit (Tiangen Biotech Co., Beijing, China). We constructed a paired-end genome library with an insert size of 300 bp, which was further sequenced on Illumina Hiseq 2500 platform (Illumina Inc., San Diego, CA). Approximately 2.24 Gb of sequence data were generated. After trimming and filtering through Trimmomatic v0.32 (Bolger et al. 2014), 2.03G clean data were *de novo* assembled into the chloroplast genome by Velvet v1.2.07 (Zerbino and Birney 2008). Annotation was conducted by tRNAscan-SE (Schattner et al. 2005) and DOGMA (Wyman et al. 2004) with default settings to identify protein-coding genes, rRNAs, and tRNAs.

The circular genome is 152,067 bp in size (Genbank accession number: MN017598) and comprises a large single copy (LSC) region (81,187 bp), a small single copy (SSC) region (21,590 bp), and two inverted repeats (IRa and IRb) regions (24,645 bp). The total GC content is 41.33%, while the corresponding values are 44.89%, 37.58%, and 40.15% in IRs, SSC, and LSC, respectively. The genome encodes 130 genes, including 88 protein-coding genes, 33 tRNA genes, eight rRNA genes, and one pseudogene. Among them, 13 genes are duplicated, including four protein-coding genes (*rps12, rps7, psbA*, and *ycf2*), five tRNA genes (*trnH-GUG, trnI-GAU, trnA-UGC, trnT-UGU*, and *trnR-ACG*) and four rRNA genes (*rrn4.5, rrn5, rrn16*, and *rrn23*). Moreover, 18 genes contain introns with 15 having one intron (*ndhB*, *rps16*, *atpF*, *rpoC1*, *petB*, *petD*, *ndhA*, *rpl16*, *rpl2*, *trnG-UCC, trnV-UAC, trnA-UGC, trnI-GAU, trnL-UAA,* and *trnT-UGU*), while three have three introns (*ycf3, clpP*, and *rps12*).

In order to assess the phylogenetic relationship among *P. amoena* and the other ferns, we selected 13 related complete chloroplast genomes with *Alsophila spinulosa* as outgroup. Their alignment was created by MAFFT (Katoh and Standley 2013) and further used to constructed Maximum Likelihood (ML) phylogenetic tree through RAxML with 1000 replicates (Stamatakis 2014). The result reveals that *P. amoena* is clustered with polypodiaceous fern with high bootstrap values (Figure 1). This study provides new genomic resources to resolve taxonomic disputes of Polypodiaceae.

## Figures and Tables

**Figure 1.**
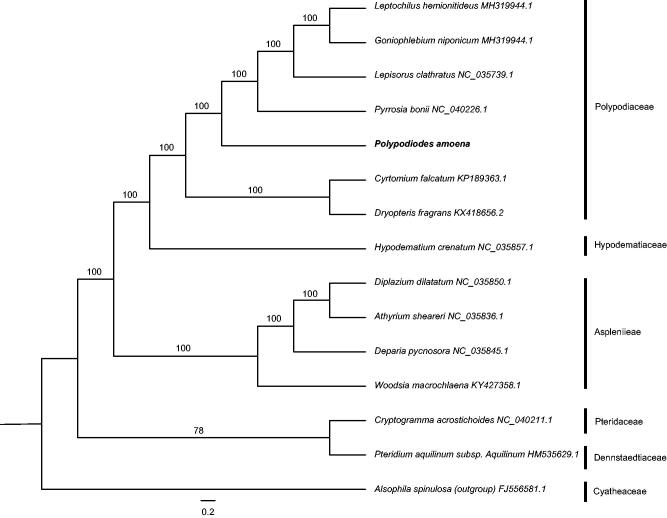
Maximum-likelihood (ML) analysis of *P. amoena* and other related ferns based on the complete chloroplast genome sequence with *Alsophila spinulosa* as outgroup. Numbers in the nodes exhibit bootstrap support values.
